# Molecular responses of human skin to single and repeated low-dose solar-simulated UVR exposure

**DOI:** 10.1016/j.xjidi.2026.100551

**Published:** 2026-10-08

**Authors:** Michael Peake, Kirsty J. Rutter, Nathan J. Hawkshaw, Mark D. Farrar, Lesley E. Rhodes

**Affiliations:** 1 Centre for Dermatology Research, Division of Musculoskeletal and Dermatological Sciences, School of Biological Sciences, Faculty of Biology, Medicine and Health, NIHR Manchester Biomedical Research Centre, The University of Manchester, Manchester, United Kingdom; 2 Photobiology Unit, Dermatology Centre, Salford Royal Hospital, Northern Care Alliance NHS Foundation Trust, Manchester Academic Health Science Centre, Manchester, United Kingdom

**Keywords:** Cell proliferation, Gene expression profiling, IFNs, Long noncoding RNA, RNA-seq

## Abstract

Solar UVR significantly influences human health, yet the early molecular effects of everyday low-dose exposure remain poorly understood. This study investigated acute molecular events in human skin after single and repeated low-dose UVR exposure, providing insights into early regulatory networks underlying cutaneous UVR responses. Healthy volunteers (n = 6, 5 female, aged 28–65 years, skin types II–III) received low-dose solar-simulated UVR to upper buttock skin on 3 consecutive days, with biopsies collected 30 minutes, 3 hours, and 24 hours after the 1st exposure; 24 hours after the 3rd exposure; and from unexposed skin. Biopsies were assessed by immunostaining and RNA sequencing. Whereas a single exposure induced few transcriptomic changes, repeated UVR exposure elicited a robust response with 1498 differentially expressed genes compared with those in unexposed skin. This included numerous long noncoding RNAs and showed pathway enrichment related to cell-cycle activation, inflammation, and IFN signaling. Accompanying this was the activation of upstream regulators, including IFNs, GFs, and proinflammatory molecules. Immunostaining suggested an apparent (nonsignificant) increase in epidermal cell proliferation and immune infiltration after 3 low-dose UVR challenges. The findings demonstrate that even brief repeated UVR exposures can induce cumulative effects, evoking molecular responses relevant to skin physiology.

## Introduction

UVR in sunlight is a major environmental factor that influences human skin health. The best-recognized benefit of UVR exposure is the cutaneous synthesis of vitamin D, a crucial hormone involved in calcium and phosphate homeostasis and bone mineralization (Lips and van Schoor, 2011). Solar UVR exposure is also associated with other aspects of health, which may be conveyed by vitamin D–dependent or vitamin D–independent means, including protection against certain autoimmune diseases and infections (Frandsen et al, 2014; Neale et al, 2023; Penckofer et al, 2010). However, UVR exposure can be detrimental to the skin, leading to oxidative stress, immunosuppression, and the formation of cytotoxic DNA lesions, primarily cyclobutane pyrimidine dimers. The latter can result in sequence alterations that initiate carcinogenesis if they are not effectively resolved through nucleotide excision repair or apoptosis (Pfeifer, 2020; Rastogi et al, 2010). Excessive UVR exposure causes acute inflammation of the skin, manifesting clinically as sunburn erythema and accompanied histologically by neutrophil and T-lymphocyte accumulation (Bernard et al, 2019; Hawkshaw et al, 2020). Adaptive responses occur, including melanogenesis triggered by UVR-induced DNA damage and epidermal thickening after keratinocyte proliferation (El-Abaseri et al, 2005).

Assessing the skin transcriptomic response to controlled UVR exposures provides insights into early molecular mechanisms that may be engaged during responses to natural sunlight. Previous in vivo work has explored the response of the human skin transcriptome to a single moderate-high dose of UVR (Bustamante et al, 2020). After 3 and 6 standard erythema doses of solar-simulated UVR (equivalent to approximately 1 and 2 minimal erythema doses [MEDs], respectively, for an individual of Fitzpatrick skin type II), dose-dependent transcriptional changes were observed, peaking 6 hours after exposure, with early transcriptional responses primarily relating to apoptosis and keratinization. After 24 hours, genes associated with inflammatory responses were predominant (Bustamante et al, 2020). Another human study explored epidermal transcriptomic and epigenetic changes in blister roofs after near-erythemal UVR doses (0.9 of each individual’s MED); the epidermis was separated from the dermis using suction, which involves some trauma (Holzscheck et al, 2020). Substantial interindividual variation in UVR responses was observed, with distinct molecular phenotypes observed to be enriched for DNA repair, immune signaling, stress response, and metabolic pathways (Holzscheck et al, 2020). In vitro, UVA and UVB induced both shared and distinct gene/protein expression signatures in human keratinocytes, including pathways linked to keratinization, lipid metabolism, and stress responses, highlighting the importance of examining the combined effects of UVB and UVA, as would be experienced from sunlight exposure (Karisola et al, 2024; Zhao et al, 2023).

Despite advances in understanding the effects of higher doses of UVR on the skin, the early molecular events triggered by low-dose, repeated UVR exposures (mimicking incidental, everyday sun exposure in humans) remain little explored. Importantly, such brief, repeated exposures are a public health recommendation for people to “safely” gain vitamin D benefits from sunlight (Cancer Research UK, 2024). Therefore, the main objective of this study was to investigate the acute molecular responses of the skin to low-dose, solar-simulated UVR exposures, both over a time course after a single exposure and after repeated exposures, in healthy humans. Cellular changes and biophysical skin measurements were examined alongside.

## Results

### Study participants and biophysical skin measurements

Six healthy participants were recruited to the study (Table 1). Skin biopsies were obtained from UVR-exposed and -unexposed skin for cellular and molecular analyses. Biopsies from 4 participants were processed for RNA sequencing. Biophysical measurements taken 24 hours after a single low-dose UVR exposure (1× UVR) and after 3 consecutive daily low-dose UVR exposures (3× UVR) suggested small increases in transepidermal water loss and pigmentation (nonsignificant) and a small, significant (*P* < .05) increase in skin redness (a∗) after 3× UVR (Figure 1).

**Table 1:** Baseline Characteristics of Study Participants

Participant	Age, y	Sex	FST	ITA^o^	TEWL (g/m^2^/h)	a∗
1	47	Female	II / III	55.7	5.8	7.0
2	28	Male	II	56.4	8.0	4.5
3	60	Female	III	26.5	7.9	9.4
4	65	Female	III	58.3	5.5	5.6
5	30	Female	II / III	52.2	8.2	4.6
6	40	Female	II	54.1	6.6	4.5

Abbreviations: FST, Fitzpatrick skin type; ITA, individual typology angle; TEWL, transepidermal water loss.

**Figure 1:**
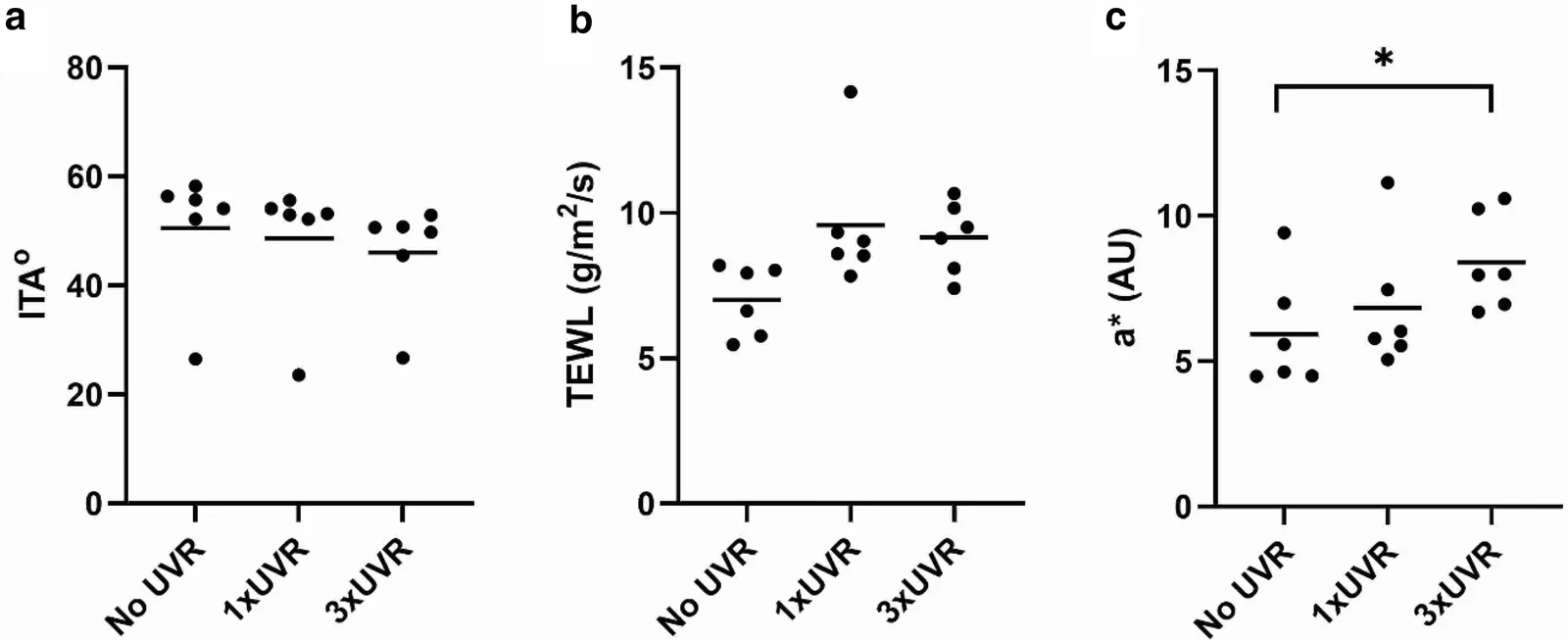
**Biophysical measures.(a)** ITA^o^, **(b)** TEWL, and **(c)** redness (a∗) of unexposed skin (no UVR) and skin 24 hours after exposure to a single (1× UVR) or 3 consecutive daily (3× UVR) doses of UVR. Individual data and mean (horizontal bars) are presented; n = 6; ∗*P* < .05. ITA, individual typology angle; TEWL, transepidermal water loss.

### Consecutive daily low-dose UVR exposure induces greater differential gene expression than a single exposure

Compared with unexposed skin, 1× UVR induced a time-dependent increase in differential gene expression, with 15 genes upregulated and 12 downregulated 30 minutes, 41 upregulated and 46 downregulated 3 hours, and 25 upregulated and 112 downregulated 24 hours after exposure. However, 24 hours after 3× UVR, differential gene expression increased substantially, with 720 genes upregulated and 778 downregulated. Only a single gene, *KAP6-1*, was significantly upregulated at all time points after 1× UVR and also after 3× UVR (Figure 2a and b). Of the 137 differentially expressed genes (DEGs) identified 24 hours after 1× UVR, 74 were shared with those identified after 3× UVR, contrasting with earlier time points after 1× UVR, where shared DEGs were minimal (<10). Notably, 1409 DEGs were unique to the 3× UVR challenge (Figure 2a). The top 20 DEGs, ranked by adjusted *P*-value, included genes associated with melanogenesis (*PMEL*, *TYR*, *TYRP1*), cell-cycle regulation (*CDK2AP2*, *STARD9*, *ZBTB7A*, *EPIC1*), and cellular stress (*HSPB1*, *PRKN*) (Figure 2b and Table 2). Furthermore, 333 DEGs identified after 3× UVR were long noncoding RNAs, accounting for 22.4% of all DEGs identified after this multiple UVR challenge (Figure 2c).

**Figure 2:**
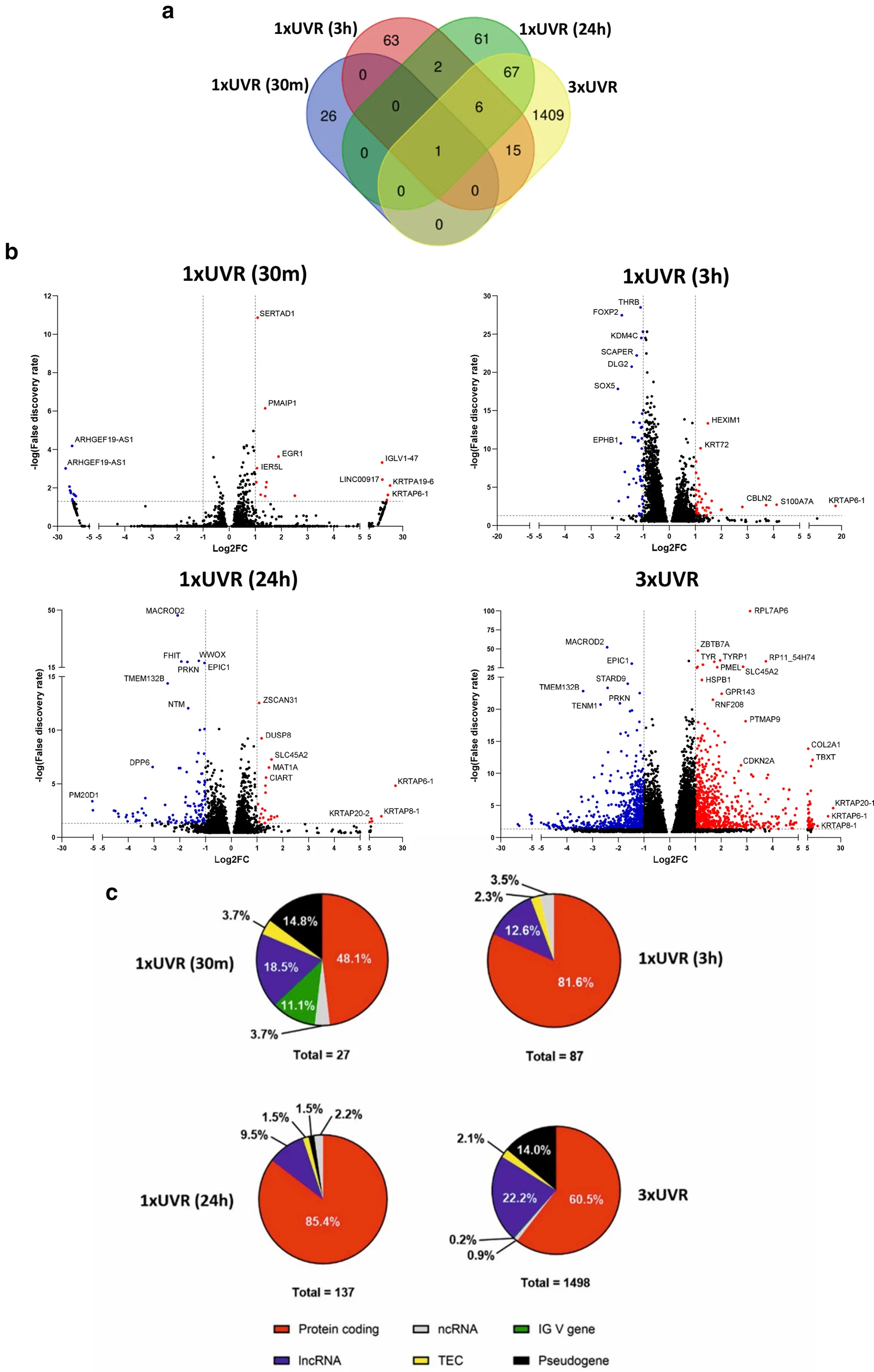
**Transcriptomic analysis of healthy human skin exposed to low-dose UVR compared with unexposed skin.(a)** Venn diagram showing the number of unique and overlapping DEGs at different time points after 1× UVR and after 3× UVR compared with those in unexposed skin. **(b)** Volcano plots of DEGs for post-UVR versus unexposed skin. Red indicates upregulated genes, blue indicates downregulated genes, and black indicates genes that did not meet predefined filter thresholds. **(c)** Distribution of DEGs by gene category for each time point and challenge; n = 4. DEG, differentially expressed gene; IG V, variable chain immunoglobulin gene; long noncoding RNA; ncRNA, noncoding RNA, lncRNA, TEC, to be experimentally confirmed.

**Table 2:** Top 20 DEGs (by q-Value) in the Skin after 3× UVR Versus Unexposed Skin

Gene	Expr False Discovery Rate (q-Value)	Expr Log Ratio (Log_2_FC)
*RPL7AP6*	1.95E-100	3.126
*MACROD2*	7.02E-53	−2.434
*ZBTB7A*	1.81E-48	1.098
*TYRP1*	1.30E-35	1.962
*RP11_54H74*	1.99E-34	3.738
*TYR*	9.19E-34	1.734
*EPIC1*	2.81E-31	−1.473
*CDK2AP2*	6.45E-30	1.291
*SLC45A2*	2.99E-27	2.855
*ENTREP3*	5.30E-27	1.085
*PMEL*	1.43E-26	1.851
*SNRPA*	9.09E-26	1.055
*HSPB1*	2.79E-25	1.255
*STARD9*	1.05E-24	−1.63
*FPGT-TNNI3K*	4.76E-24	−2.413
*TMEM132B*	1.51E-23	−3.367
*DNAH1*	3.08E-23	−1.165
*GPR143*	3.91E-23	2.023
*RNF208*	3.33E-22	1.68
*PRKN*	1.22E-21	−1.938

Abbreviations: DEG, differentially expressed gene; FC, fold change.

### Repeated low-dose UVR exposure activates proinflammatory, IFN, and cell-cycle–associated upstream regulators within the skin

A number of upstream regulators (URs) were predicted to be activated after 3× UVR (Table 3), the majority of which were not predicted to be activated after 1× UVR. This included proinflammatory mediators such as TNF and IL-1B; transcription factors and GFs, including VEGF, hepatocyte GF, and microphthalmia-associated transcription factor; and IFN signaling–associated URs, including IFN-γ, signal transducer and activator of transcription 1, IFN-λ1, and IFN regulatory factor 7 (Table 3). The UR with the highest activation score after 3× UVR was EGFR long noncoding downstream RNA (ELDR), which is implicated in cell-cycle regulation and was predicted to be activated on the basis of the differential expression of cell-cycle regulation genes, including *AURKA*, *AURKB*, *CDC25C*, *CCNB1*, *PLK1*, and *CDK1* (Figure 3).

**Table 3:** Top 20 Upstream Regulators Predicted to Be Activated after 3× UVR Compared with those in Unexposed Skin

Upstream Regulator	Expr Log Ratio (Log_2_FC)	Molecule Type	Activation z-score	*P-*Value of Overlap
ELDR	—	Other	6.601	2.07E-51
HGF	−0.773	Growth factor	5.821	1.86E-10
IFN-γ	2.519	Cytokine	5.779	1.37E-09
CSF2	0.121	Cytokine	5.694	3.43E-11
MITF	0.217	Transcription regulator	5.535	1.60E-14
NONO	0.285	Transcription regulator	5.514	1.66E-15
CKAP2L	1.201	Other	5.303	3.42E-32
VEGF (family)	—	Group	5.287	5.46E-13
CEBPB	1.227	Transcription regulator	5.284	2.63E-19
TFEB	0.615	Transcription regulator	5.151	7.11E-08
IRF7	1.654	Transcription regulator	5.145	1.26E-10
IFN-λ1	−0.981	Cytokine	5.109	8.78E-18
ERBB2	0.618	Kinase	5.079	6.85E-11
STAT1	1.659	Transcription regulator	5.015	1.56E-14
IFN-α2	—	Cytokine	4.942	3.33E-13
IFN-α (family)	—	Group	4.847	8.03E-14
TNF	0.344	Cytokine	4.823	5.06E-07
IFN-β (family)	—	Group	4.767	1.66E-15
FOXM1	1.959	Transcription regulator	4.686	1.68E-18
CHROMR	−0.105	Other	4.642	2.66E-15

Abbreviations: HGF, hepatocyte GF; IRF7, IFN regulatory factor 7; MITF, microphthalmia-associated transcription factor.

**Figure 3:**
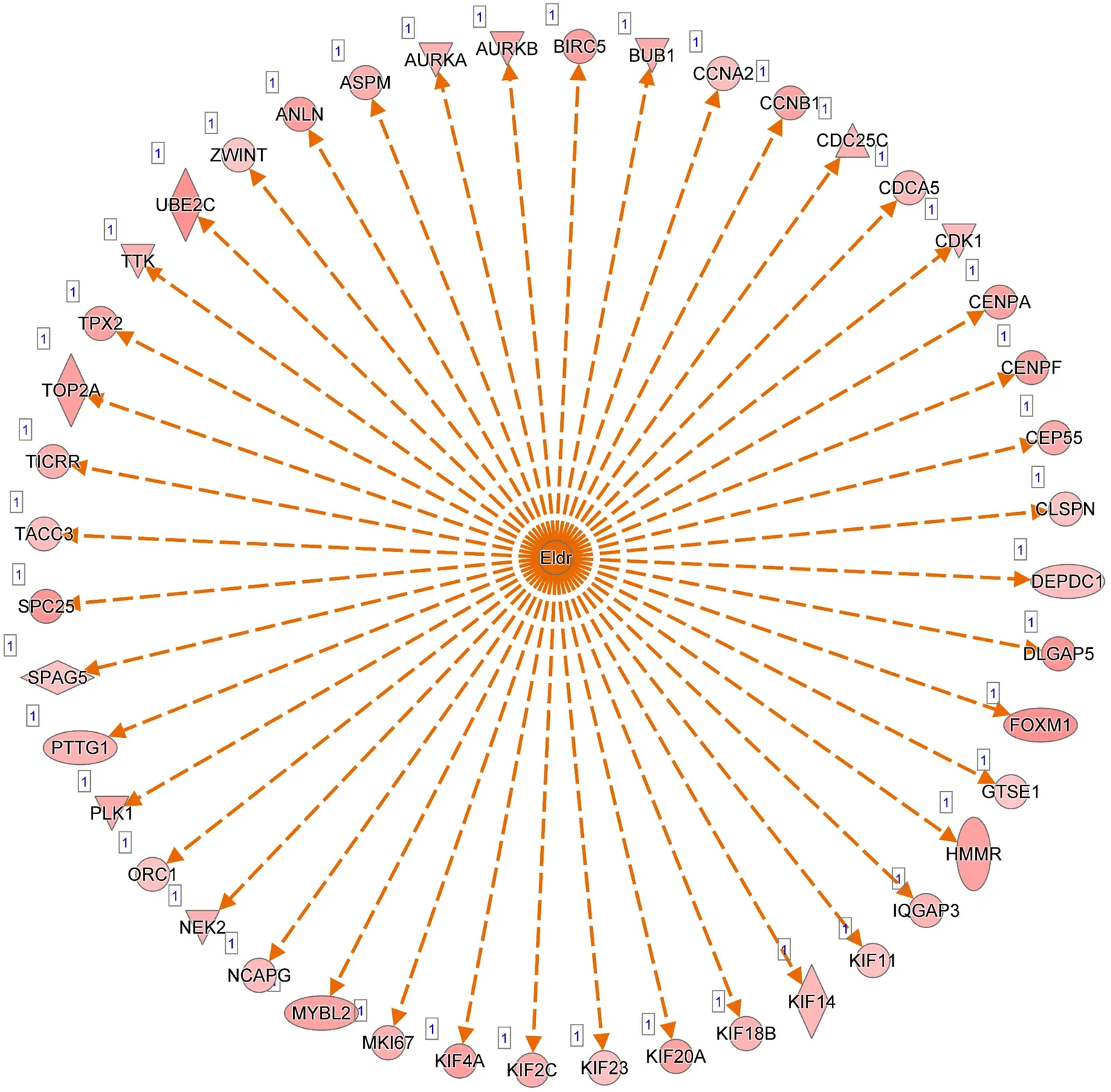
**ELDR is the top predicted upstream regulator after repeated low-dose UVR.** Shown are differentially expressed genes predicting activation of the upstream regulator ELDR. ELDR, EGFR long noncoding downstream RNA.

### Repeated daily low-dose UVR resulted in enrichment of pathways linked to cell-cycle regulation and immune responses

Canonical pathway analysis identified enriched pathways after 3× UVR (Figure 4a) that were not enriched at any time point after 1× UVR. These included pathways associated with cell-cycle regulation, such as the activation of the kinetochore metaphase signaling pathway and the inhibition of the cell-cycle G2/M DNA damage checkpoint regulation pathway. Immune response pathways, such as IFN signaling and pathogen-induced cytokine storm signaling, were also activated. The DEGs associated with the predicted inhibition of the cell-cycle G2/M DNA damage checkpoint regulation did not exhibit significant differential expression after 1× UVR (Figure 4b). In view of the predicted cell-cycle upregulation after 3× UVR, staining for the cell proliferation marker Ki-67 (Scholzen and Gerdes, 2000) was performed (Figure 4c). The mean number of Ki-67–positive cells observed after 3× UVR was higher than after 1× UVR and in unexposed skin, but it did not reach statistical significance **(**Figure 4d). Mean epidermal thickness after 1× UVR or 3× UVR was unchanged compared with that in unexposed skin (Figure 4e).

**Figure 4:**
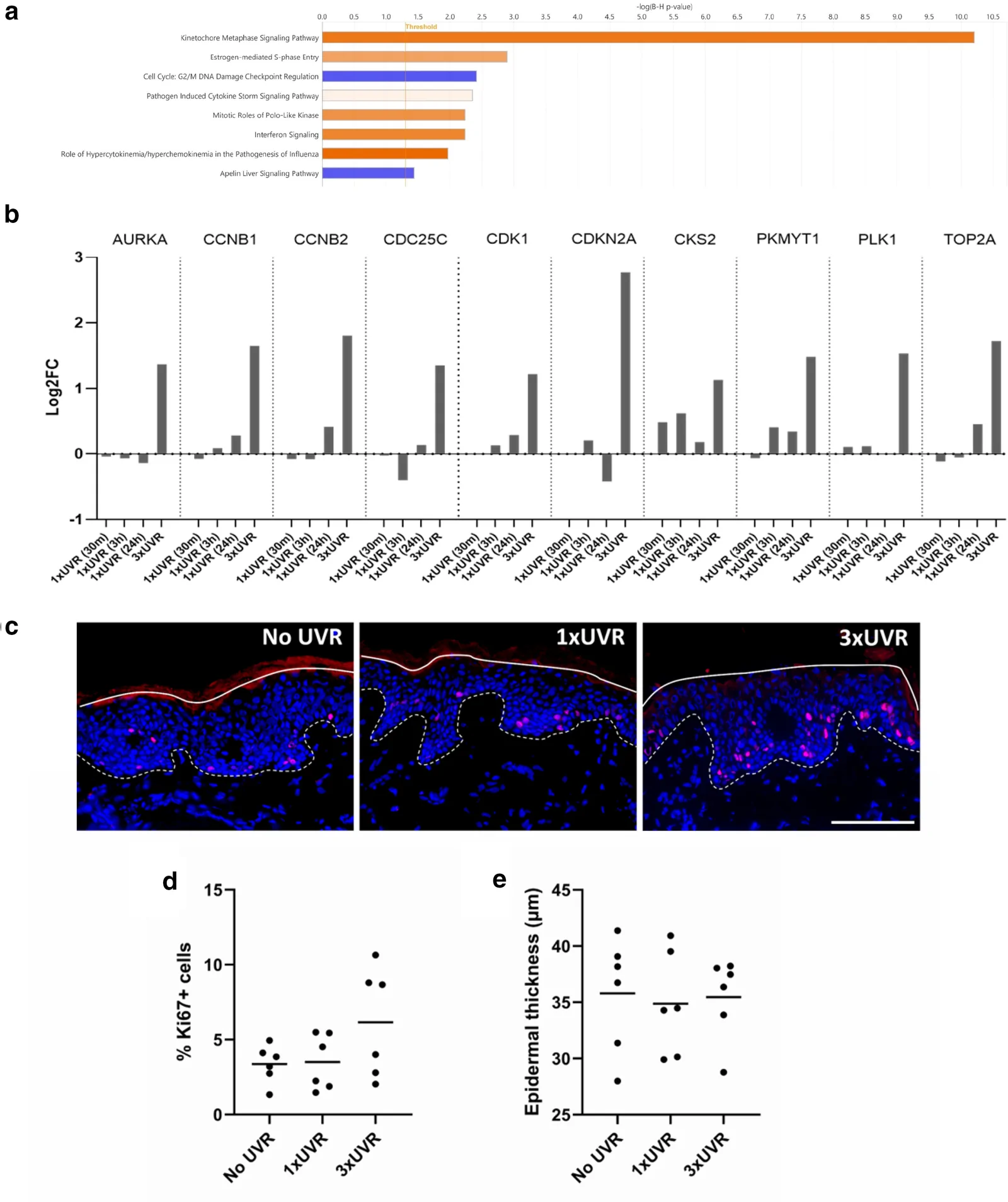
**Canonical pathways, cell-cycle damage checkpoint-associated genes, and epidermal proliferation assessment.(a)** Top 8 enriched pathways after 3 consecutive daily UVR exposures (3× UVR). Values are −log of Benjamini–Hochberg–corrected *P*-values, where the level of significance reflects the unlikelihood that the association of molecules from this dataset with the canonical pathway occurs by random chance. Orange bars denote predicted activation, and blue bars denote inhibition. **(b)** Log_2_FC of DEGs associated with the cell-cycle G2/M DNA damage checkpoint regulation pathway after 1× UVR and 3× UVR. **(c)** Representative images of epidermal Ki-67 staining in unexposed skin (no UVR) and skin 24 hours after exposure to 1× UVR or 3× UVR. Bar = 100 μm. **(d)** Percentage of epidermal cells expressing Ki-67, and **(e)** inter**-**ridge epidermal thickness of unexposed skin (no UVR) and skin 24 hours after exposure to 1× UVR or 3× UVR. Individual data and mean (horizontal bars) are presented; n = 6. DEG, differentially expressed gene; FC, fold change.

### Repeated low-dose UVR exposure may induce low-level immune cell infiltration

Because canonical pathways related to inflammatory responses were enriched after repeated doses of UVR, potential immune infiltration was investigated. First, the relative abundance of immune cells was estimated from gene expression data using CIBERSORT. This suggested a shift in the proportions of immune cells after 3× UVR compared with that after 1× UVR and to unexposed skin, notably an increase in the proportion of CD4+ T cells and in activated dendritic cells (Figure 5), although these did not reach statistical significance. To investigate this further, we performed immunofluorescence staining of skin biopsy sections to identify CD4+ T cells and CD11c+ dermal dendritic cells. An increase in both cell types was observed after 3× UVR compared with that in unexposed skin, but the differences were not significant, and there was substantial interindividual variance (Figure 6).

**Figure 5:**
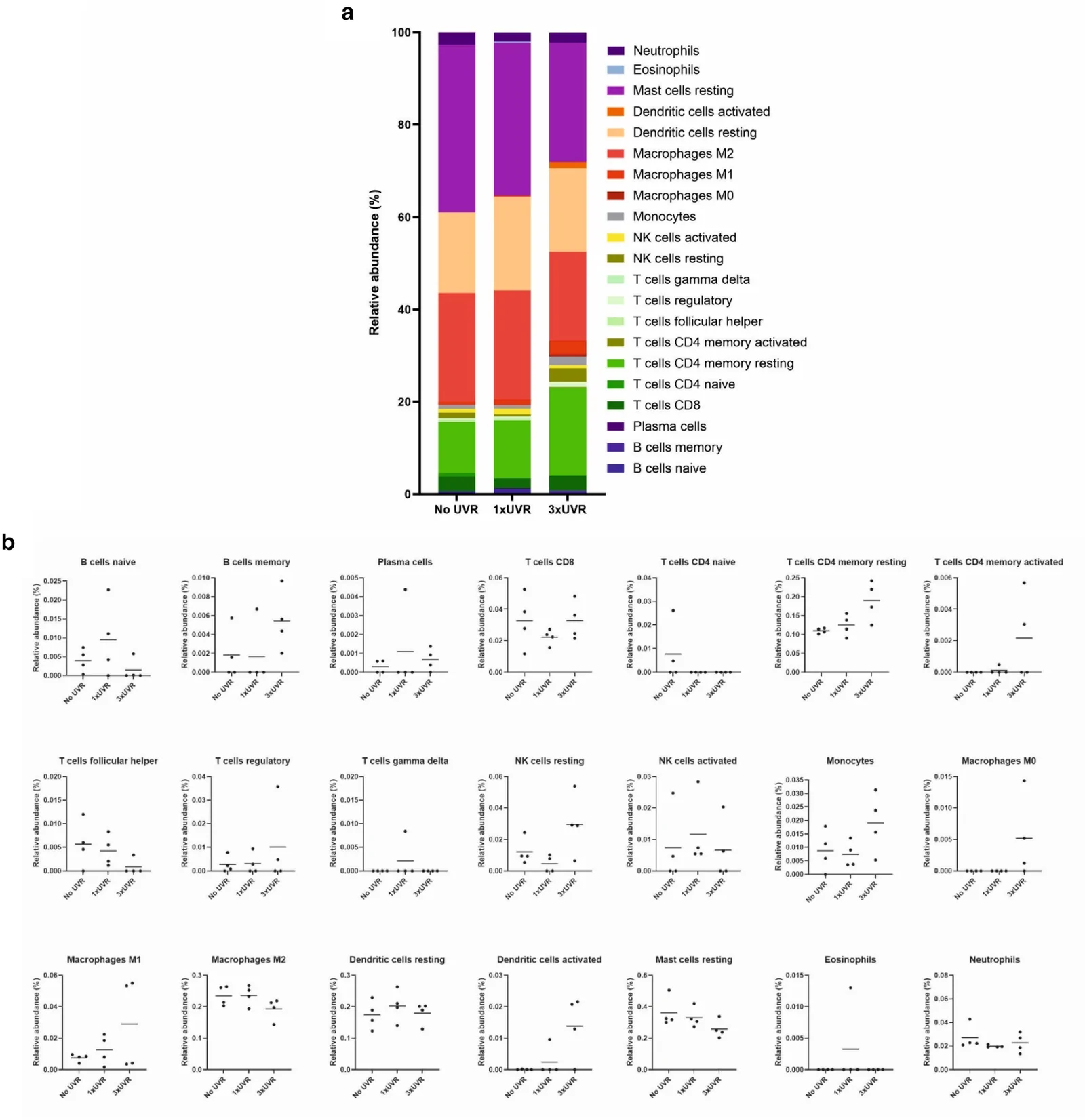
**Transcriptomic assessment of dermal immune cell infiltration.(a)** Compiled and **(b)** individual prediction of cell-type relative abundance in the immune infiltrate using CIBERSORT software in unexposed skin (no UVR) and 24 hours after 1× UVR or 3× UVR.

**Figure 6:**
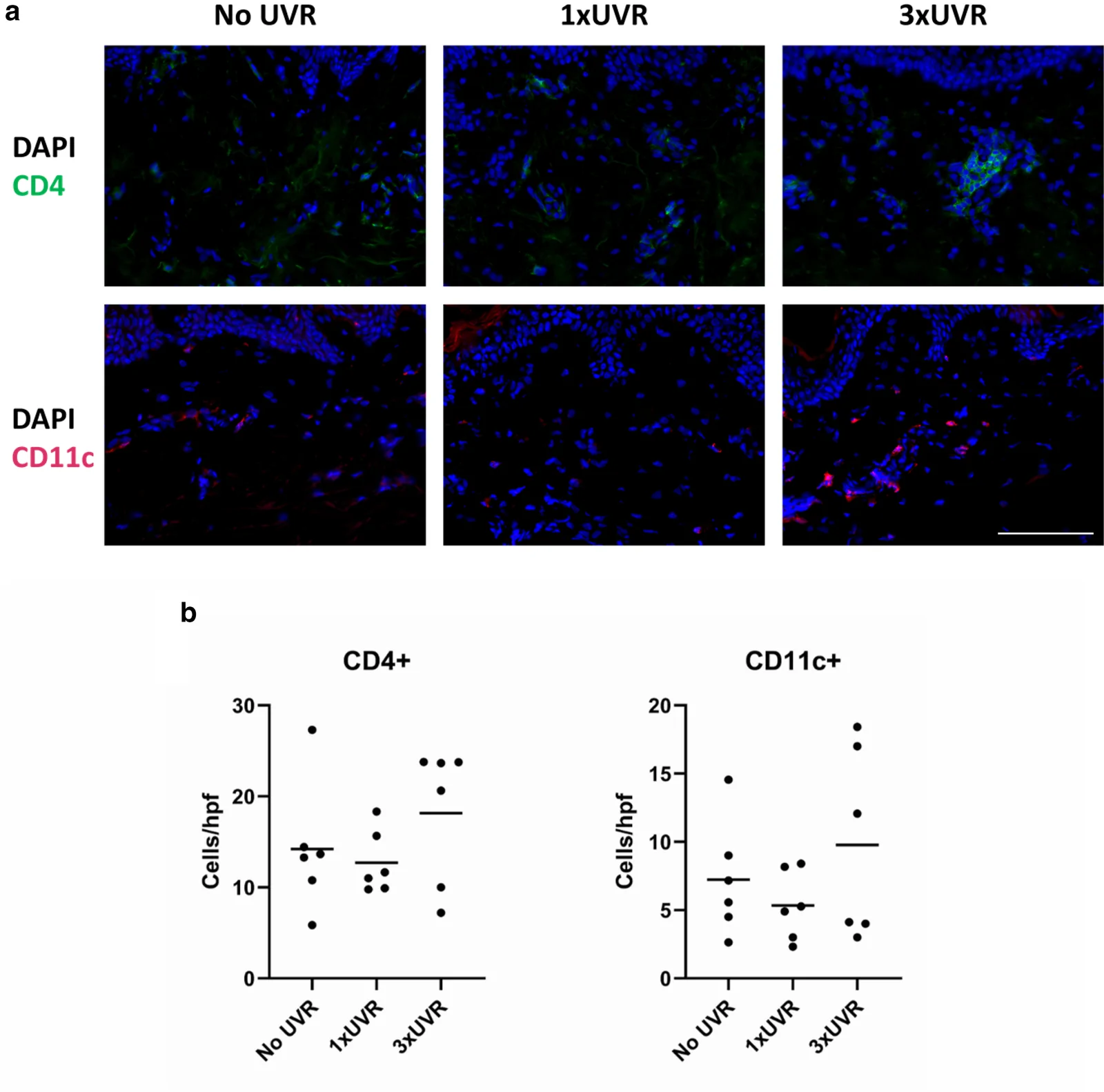
**Immunofluorescent assessment of dermal immune cell infiltration.(a)** Representative immunostaining images and **(b)** quantification of CD4+ T cells and CD11c+ dermal dendritic cells in unexposed skin (no UVR) and skin 24 hours after exposure to 1× UVR or 3× UVR. Bar = 100 μm. Individual data and mean (horizontal bars) are presented; CD4+, n = 6; CD11c+, n = 5.

## Discussion

We investigated the effect of single and repeated low-dose UVR exposure, as may occur incidentally in everyday life, on the transcriptomic and related cellular responses of skin in healthy humans. Evaluation of whole skin (epidermis and dermis) demonstrated that a single low-dose UVR exposure mediated few changes in the transcriptional profile, with only a small increase in the number of DEGs from 30 minutes to 24 hours after exposure. However, 3 consecutive daily exposures led to a striking increase in the number of DEGs that was accompanied by a more pronounced cellular response in the skin. These findings contrast with those of Bustamante et al (2020), where 6 hours after exposure to a single higher UVR dose yielded the highest number of DEGs, followed by a reduction at 24 hours. This likely reflects the different experimental designs, with Bustamante et al (2020) using higher doses of 3 and 6 standard erythema doses (approximately 1 and 2 MEDs, respectively, for an individual of Fitzpatrick skin type II) compared with the 2 standard erythema doses used in our study. Their higher dose induced greater acute transcriptional changes associated with apoptosis and keratinization at 6 hours, responses that were not observed in our study, followed by inflammatory and immune signaling pathways at 24 hours, consistent with a more pronounced erythemal response. In contrast, our findings showed that although a single low-dose UVR exposure induced minimal transcriptional changes, the skin did not fully return to its pre-exposure transcriptional state within 24 hours, and subsequent UVR exposures produced a cumulative effect.

Transcriptomic studies in murine skin have also demonstrated dose- and time-dependent responses after UVR exposure, including activation of pathways associated with DNA damage responses, inflammation, oxidative stress, and epidermal remodeling (Qutob et al, 2024). Although the erythemally weighted UVR dose used in this mouse study included a comparable 20 mJ/cm^2^ exposure, the magnitude and nature of the transcriptional responses differ from those observed here. This likely reflects species-specific differences in epidermal structure, immune regulation, and UVR responsiveness and illustrates the importance of validating findings in humans.

Our transcriptomic data suggest that repeated low-dose UVR exposure may stimulate epidermal keratinocytes to initiate cell-cycle entry and division, consistent with the activation of proliferative pathways. This response was not observed after a single exposure. No significant change in epidermal thickness was seen, which suggests that although a transcriptional program consistent with proliferative activation had been initiated, it had not yet translated into a measurable structural change within the epidermis. Upregulation in epidermal cell proliferation after UVR exposure has been reported in both mouse models (El-Abaseri et al, 2005) and human skin, including skin substitutes and in vivo (Lee et al, 2002; Michalczyk et al, 2018). Notably, an early study by Lee et al (2002) investigated the effects of sunburn-inducing doses of UVB (1–3 MEDs) on the buttock skin of healthy volunteers, analyzing samples 24, 48, and 72 hours after exposure. Their findings demonstrated a dose-dependent increase in Ki-67 expression peaking at 48 hours, with no significant differences observed at 24 or 72 hours. In addition, repetitive exposure to 1 MED for 5 consecutive days led to a more pronounced increase in Ki-67 expression than a single exposure (Lee et al, 2002). These findings highlight UVR-induced photoadaptive changes that align with our results, whereas our study demonstrates that even low, individually subsunburn dose exposures over just 3 days may be sufficient to initiate early proliferative signaling in the epidermis.

A potential mechanism for the observed epidermal proliferation is through the activation of the EGFR (El-Abaseri et al, 2005), which is expressed by keratinocytes (Joly-Tonetti et al, 2021; Mascia et al, 2010). Transcriptomic changes predicted activation of the ELDR, which was the top UR in response to repeated low-dose UVR exposure. ELDR may play a regulatory role in epidermal cell proliferation, as suggested by the cell-cycle–associated DEGs that contributed to the overall prediction of ELDR activation (Figure 3). This aligns with a previous in vitro study of oral keratinocytes, which demonstrated that ELDR promotes cell-cycle progression through the CTCF–FOXM1–AURKA signaling axis (Sur et al, 2022). Genes within this axis, for example, *AURKA* and *FOXM1*, were also significantly upregulated in our study after 3× UVR. ELDR has also been suggested as a potential oncogene owing to its overexpression (as well as EGFR) in different cancer tissues (El-Abaseri and Hansen, 2007; Sur and Ray, 2022). Although other long noncoding RNAs have been identified to play roles in the development of skin cancer and are differentially expressed in malignancies such as melanoma compared with healthy tissues (Sand et al, 2016; Wozniak and Czyz, 2021), ELDR has not been implicated in skin cancer. Our finding supports the need for further research to elucidate the potential role of ELDR in cancer progression and skin biology, especially UVR responses.

We found that repeated low-dose UVR upregulated melanogenesis genes *PMEL*, *TYR*, and *TYRP1*, and activation of the UR microphthalmia-associated transcription factor was predicted. This upregulation was not observed after a single UVR exposure. This is consistent with prior protein-based analyses of melanogenesis markers after repeated UVR exposure, which have demonstrated that melanogenesis and subsequent tanning responses are induced by low, repetitive doses of UVR (0.4–0.5 MED; 5 times a week for 2 weeks) (Choi et al, 2010). The induction of melanogenic transcripts after 3× UVR likely reflects an initial molecular phase of pigmentation that precedes the measurable increases in melanin content reported with extended low-dose UVR regimens (de Boer et al, 2025). Furthermore, these findings may indicate the occurrence of acute skin injury, which in turn stimulates the melanogenic response. It is reported that DNA damage can occur at very low doses of UVR, down to 0.2 MED (Shih et al, 2018). Acute damage could also be mediated by the accumulation of ROS, because cumulative UVR exposure can overwhelm ROS defense mechanisms and subsequently increase oxidative stress (Panich et al, 2016). In addition, our analysis predicted activation of VEGF after 3× UVR, which has been identified as an immediate-early activator in UVR-induced skin damage (Hartono et al, 2022).

Repeated UVR exposure led to the enrichment of IFN signaling pathways and predicted upstream activation of IFNG, IFNL1, and IFNA2, indicating potential proinflammatory responses involving IFN. Studies in mice have shown that UVB exposure can stimulate IFN signaling in the skin (Tam et al, 2019). We propose that the observed IFN signaling here is primarily associated with the regulation of apoptotic pathways, particularly exerting its reported antiapoptotic effects (Kotredes and Gamero, 2013). Within this pathway, genes such as *STAT1* and *IFIT3*, which have been shown to promote cell survival and display antiapoptotic functions, were significantly upregulated compared with those in unexposed skin.

Excessive UVR exposure induces an inflammatory response (ie, sunburn) in the skin, characterized by infiltration of neutrophils and lymphocytes and erythema (Di Nuzzo et al, 1998; Rhodes et al, 2001; Strickland et al, 1997). We found that a single lower dose of UVR exposure did not result in any significant change in skin redness (a∗) and that there was no evidence of immune cell infiltration when transcriptional data were deconvoluted using CIBERSORT. Immunostaining and cell quantification showed no difference in the numbers of dermal CD4+ T cells or CD11c+ dendritic cells compared with those in unexposed skin. This suggests that the administered dose of UVR was insufficient to elicit acute inflammatory responses. In contrast, skin exposed to 3× UVR demonstrated enrichment of proinflammatory pathways and, although not reaching statistical significance, suggested increases in the proportion of CD4+ T cells and CD11c+ dendritic cells predicted by CIBERSORT and in mean cell counts after immunostaining. This was accompanied by a significant increase in skin redness (a∗) and suggests a cumulative effect of UVR on the skin, whereby repeated daily suberythemal exposures may contribute to low-level immune infiltration and initiation of an erythemal response, driving the skin toward a proinflammatory state with potential implications for skin health and aging (Ansary et al, 2021). The response seen after 3× UVR may be sufficient to stimulate a subsequent counteracting immunosuppressive state (Ireland et al, 2025; Salminen et al, 2022), but this requires further investigation.

Study strengths include the use of standardized sampling procedures, where the time course of tissue collection was carefully controlled; the use of low-dose solar-simulated UVR that closely approximated real-world solar UVR exposure; and performance directly in humans in vivo. Limitations include that although this study included a detailed analysis of RNA-sequencing data with comparisons under different conditions of UVR exposure, it did not provide information on the functional output of gene expression. However, immmunohistochemistry and other assessments were performed, some of which support the RNA-sequencing findings. This study had a relatively small cohort size and a broad age range of participants (28–65 years). Future studies in larger cohorts may define the relative contribution of demographic factors to UVR-induced skin responses.

To our knowledge, we have provided previously unreported insights into the transcriptional profile of healthy human skin after low-dose UVR exposure, as obtained in daily life. We show that repeated, in contrast to single, low doses of UVR induce a substantial transcriptional response compared with that in unexposed skin, accompanied by increased epidermal cell proliferation and mild immune infiltration (nonsignificant). Our findings provide a deeper understanding of the processes underlying skin responses to UVR and highlight how even brief, repeated UVR exposures, as recommended to the public for vitamin D acquisition, can influence the skin’s physiological status and potentially its health.

## Materials and Methods

### Volunteer recruitment

Healthy males and females aged between 18 and 65 years were recruited at the Photobiology Unit, Salford Royal Hospital, Greater Manchester, United Kingdom, between October 2018 and January 2020. Participants were excluded if they reported sunbathing, sunbed use, or phototherapy in the past 3 months; were taking photosensitizing or immunosuppressive medication; or had a history of skin cancer. All participants gave written informed consent.

### Study protocol

A 5 × 5 cm area of upper buttock skin was exposed on 3 consecutive days to 10 J/cm^2^ of solar-simulated UVR (total unweighted UVR, 290–400 nm) using a 1 kW xenon arc lamp plus atmospheric attenuation filter (Newport Spectra-Physics). This dose approximates to 20 mJ/cm^2^ erythemally weighted UVR (2 standard erythema doses), equivalent to ∼30 minutes exposure to midday summer sunlight, unshaded and unprotected, on a clear day (United Kingdom). Skin punch biopsies (5 mm in diameter) were taken from unexposed skin and from UVR-exposed skin 30 minutes, 3 hours, and 24 hours after a single UVR exposure (1× UVR) and 24 hours after the third of 3 consecutive daily UVR exposures (3× UVR). Biopsies were bisected perpendicular to the epidermal surface; then, 1 half was placed in optimal cutting temperature compound (CellPath, Powys, United Kingdom), snap frozen in liquid nitrogen, and stored at −80 °C. The other half was placed in RNAlater stabilization solution (Thermo Fisher Scientific, Paisley, United Kingdom) at 4 °C for at least 48 hours; then, the solution was removed, and the biopsy was stored at −80 °C.

### Biophysical skin measurements

Skin pigmentation (ITA^o^ [individual typology angle]) and redness (a∗) were quantified using a reflectance spectrophotometer (CM-600d, Konica Minolta) and the CIE L∗a∗b∗ color space (Ly et al, 2020). Skin transepidermal water loss was measured using a Vapometer (Delfin Technologies, Kuopio, Finland) (Akdeniz et al, 2018; Alexander et al, 2018) after acclimatization for 15–20 minutes at a mean room temperature of 21 °C and a humidity of 52% (within the recommended levels) (Alexander et al, 2018; Pinnagoda et al, 1990; Rogiers, 2001). All measurements were taken in triplicate at each site.

### RNA extraction, sequencing (RNA sequencing), and analysis

The RNeasy Plus Universal Minikit (Qiagen, Manchester, United Kingdom) was used to extract RNA from biopsies per the manufacturer’s instructions. RNA quantity and integrity were verified using the Agilent 2200 TapeStation (Agilent Technologies, Santa Clara, CA). For RNA sequencing, data quality checking, and transcript identification, the HiSeq4000 system (Illumina, San Diego, CA) was used at the University of Manchester Genomics Core Technology Facility. The paired-end sequences were assessed by FastQC and FastQ Screen (http://www.bioinformatics.babraham.ac.uk/projects/fastqc/). Sequence adapters were removed, and reads were quality trimmed using Trimmomatic 0.39 (PMID: 24695404). Reads were mapped to GRCh38 (hg38) using STAR 2.5.3a (PMID: 23104886), and gene counts were calculated using GENCODE 31 annotation. Normalization, principal components analysis, and differential expression were performed in DESeq2 1.20.0 using default settings and the design `∼ patient + condition` (PMID: 25516281), using patient as a blocking factor. Genes were filtered on the basis of log_2_-fold change thresholds of 1 and −1 with an adjusted *P*-value (q-value) of .05 to control for false discovery rate, allowing for the identification of DEGs. Transcript classification of DEGs in the form of biotype pie charts was determined using R software and the Ensembl database (https://www.ensembl.org/info/genome/genebuild/biotypes.html). Pie charts and volcano plots were created using GraphPad Prism 9 (GraphPad Software, La Jolla, CA). RNA-sequencing data were analyzed using Qiagen Ingenuity Pathway Analysis software (https://digitalinsights.qiagen.com/products-overview/discovery-insights-portfolio/analysis-and-visualization/qiagen-ipa/) (Krämer et al, 2014). Top URs were determined by z-score. In Ingenuity Pathway Analysis, URs classified as “Group” represent grouped molecular entities, such as families of related molecules, rather than individual molecular species, whereas “Other” represents regulators that do not fit within the predefined Ingenuity Pathway Analysis molecule-type categories. Exogenous chemical and pharmacological categories were excluded to focus on physiological processes. Further analysis into specific URs and associated DEGs in the form of a network map was created using the “build” function in Ingenuity Pathway Analysis. For canonical pathway analysis, Benjamini–Hochberg correction for multiple testing was applied, with −log (Benjamini–Hochberg *P*-value) >1.3 as the threshold for significance.

RNA-sequencing data were used to estimate the relative abundance of immune cell types in a mixed cell population. Gene expression profiles in the form of transcripts per million were analyzed using CIBERSORTx (https://cibersortx.stanford.edu).

### Immunofluorescence staining and microscopy

Frozen biopsies were cryosectioned at 7 μm, then air dried for 10 minutes, and fixed with 4% w/v paraformaldehyde. Cryosections were blocked in 2.5% normal horse serum (Vector Laboratories, Newark, CA). Primary antibodies were applied to cryosections overnight at 4 °C at the indicated dilutions: Ki-67 (1:500, Abcam, Cambridge, United Kingdom, ab16667), CD4 (1:50, Thermo Fisher Scientific, MA5-12259), and CD11c (1:100, Abcam, ab52632). The following day, secondary antibodies with amplifier were applied as per the manufacturer’s instructions from the VectaFluor Excel Amplified Anti-Mouse Dylight 488 Kit and VectaFluor Horse Anti-Rabbit Dylight 594 Kit (Vector Laboratories, Peterborough, United Kingdom). Slides were then mounted using Fluoroshield containing DAPI (Abcam), and fluorescence images were captured with a BX53 microscope (Olympus, Tokyo, Japan). For each parameter, 4–9 images from 2 or 3 sections at ×200 magnification were quantified using ImageJ software with manual counting.

### Statistical analysis

Data were analyzed by repeated measures 1-way ANOVA or Friedman test as appropriate using GraphPad Prism 9 (GraphPad Software) with *P* < .05 considered statistically significant.

## Ethics Statement

This human study was approved by the Greater Manchester West Research Ethics Committee and Health Research Authority with reference 18/NW/0494. The study was performed in accordance with the Declaration of Helsinki.

## Data Availability Statement

RNA-sequencing data are deposited in ArrayExpress/Biostudies with accession number E-MTAB-12218 (https://www.omicsdi.org/dataset/biostudies-arrayexpress/E-MTAB-12218).

## ORCIDs

Michael Peake: http://orcid.org/0009-0007-5525-0401

Kirsty J. Rutter: http://orcid.org/0000-0001-8048-7995

Nathan J. Hawkshaw: http://orcid.org/0000-0001-5311-3840

Mark D. Farrar: http://orcid.org/0000-0001-8602-7279

Lesley E. Rhodes: http://orcid.org/0000-0002-9107-6654

## Conflict of Interest

The authors state no conflict of interest
